# A publicly available newborn ear shape dataset for medical diagnosis of auricular deformities

**DOI:** 10.1038/s41597-023-02834-4

**Published:** 2024-01-02

**Authors:** Liu-Jie Ren, Fei Luo, Zhi-Wei Yang, Li-Li Chen, Xin-Yue Wang, Chen-Long Li, You-Zhou Xie, Ji-Mei Wang, Tian-Yu Zhang, Shuo Wang, Yao-Yao Fu

**Affiliations:** 1 FPRS Department/ENT Institute, Eye and ENT Hospital, Fudan University, Shanghai, China; 2 NHC Key Laboratory of Hearing Medicine, Fudan University, Shanghai, China; 3 Obstetrics & Gynecology Hospital, Fudan University, Shanghai, China; 4 Digital Medical Research Center, School of Basic Medical Sciences, Fudan University, Shanghai, China; 5 Academy for Engineering & Technology, Fudan University, Shanghai, China

**Keywords:** Diseases, Anatomy

## Abstract

Early and accurate diagnosis of ear deformities in newborns is crucial for an effective non-surgical correction treatment, since this commonly seen ear anomalies would affect aesthetics and cause mental problems if untreated. It is not easy even for experienced physicians to diagnose the auricular deformities of newborns and the classification of the sub-types, because of the rich bio-metric features embedded in the ear shape. Machine learning has already been introduced to analyze the auricular shape. However, there is little publicly available datasets of ear images from newborns. We released a dataset that contains quality-controlled photos of 3,852 ears from 1,926 newborns. The dataset also contains medical diagnosis of the ear shape, and the health data of each newborn and its mother. Our aim is to provide a freely accessible dataset, which would facilitate researches related with ear anatomies, such as the AI-aided detection and classification of auricular deformities and medical risk analysis.

## Background & Summary

Auricular deformity is one of the most common deformities in newborns, with an incidence of about 50%^1,2^. The strange appearance may cause mental distress, anxiety, inferiority, and interpersonal problems, and some of the severe deformities can be accompanied by hearing problems^3,4^. Auricular deformities can be treated by either plastic surgery or the non-surgical ear molding^2,5^. Ear molding is effective, costless, safe, and painless, but it should be applied within a limited time window, about 2~3 months after birth. Otherwise, the corrective effect would be hardly satisfying. In relatively severer cases when ear molding could not completely cure the deformities, ear molding is still helpful to improve the ear aesthetics, making the later surgery treatment much easier, or even unnecessary^6^. Therefore, an early diagnosis of auricular deformities is very essential for early, easy, costless, and effective interventions.

**Fig. 1:**
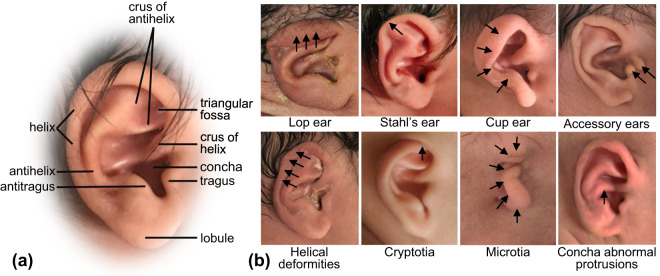
The substructures of the auricle and different types of ear deformities. (**a**) presents the morphology of a normal ear from one newborn. The ear contains multiple sub-structures. (**b**) shows examples of some sub-types of auricular deformities. The abnormal structures are marked with arrows. All the ears in this figure are from the dataset.

Diagnosis of auricular deformities heavily relies on the empirical judgement of clinicians, especially the pediatricians currently. The human auricle is quite complex with multiple sub-structures (Fig. 1a). Moreover, there are many different sub-types of auricular deformities (Fig. 1b), most commonly seen are lop ear, Stahl’s ear, cup ear, and helical deformities^1,2^. As a result, accurate identification and classification are not easy tasks, especially for less-experienced physicians.

Analyzing the auricular shape with AI is not new to the community^7^. Many studies achieved to recognize the ear location from an image of human head^8,9^. It is possible to deduce the gender and age from the shape of the ears^10,11^. Actually, the bio-metric features of the auricle are so individually specific, that make the ear shape available as another fingerprint for human recognition^12,13^. Researches have also been conducted to identify the auricular deformities and evaluate the corrective effects using convolutional neural networks^14,15^.

So far, there are quite many datasets containing the ear morphologies^7^, such as EarVN1.0^16^, UBEAR^17^, and USTB^18^ databases, etc. However, most of these datasets are for auricle detection and bio-metric feature analyzing, and most images are from adults. Although the structures of the ears of adults and babies are the same, diagnosis of baby auricular deformities can be much harder, because (1) the baby ear morphologies are more diverse than adults, and will change over time, (2) there exist some slight harmless deformations that will be self-cured, and (3) the baby ears are less distinguishable between substructures. In order to train a model to accurately identify and classify auricular deformities, a dataset of newborn babies’ ear shapes is required, along with professional diagnosis.

To address the gap, this paper describes the first release of the BabyEar4k dataset, which contains the left and right ear photos from 1,926 newborns. These photos were obtained in a controlled clinical environment, all taken with the same hardware. The image size and the proportion of the ear were controlled in order to give sufficient resolution for the ear shape. Image quality assessment were conducted for each ear image. Moreover, the diagnosis (identification and classification of deformities) from two experienced physicians were provided. Some health data of the babies and their mothers were also included. We hope that the availability of this dataset will accelerate research in image-based, computer-aided analyzing of ear morphologies, especially the identification and classification of auricular deformities in newborns.

## Methods

### Subject characteristics

The study was approved by Ethics Committee of School of Basic Medical Sciences, Fudan University. The data publication was approved by the committee, and the parents were acknowledged and provided informed consent to the open publication of the anonymized data (including the ear images and the health data). Initially, a total of 2,000 newborns between November 2018 and April 2019 were included in this study. All data were collected with the permission of the parents, being aware of the publicly open of the ear images and medical data. Inclusion criterion was viable fetus. Exclusion criterion was defined as newborns who require intensive care due to severe premature birth or other reasons. All the babies were born and taken care at the Obstetrics & Gynecology Hospital, Fudan University.

### Image acquisition, processing and quality assessment

The ear photos were taken within 48 hours after birth, a smartphone (iPhone 6 s, Apple) was used. All the photos were taken in a bright room, with no extra light sources. The newborn was placed on a flat bed, with the head on its side. Both the left and right ears were photted with a distance of about 30~60 centimeters to ensure a good resolution of the ear, and to prevent too much distortions. The camera angle was controlled by roughly aligning the camera facing the pinna, so that the substructures of the auricle could be presented. Auto focus was used mostly.

**Fig. 2:**
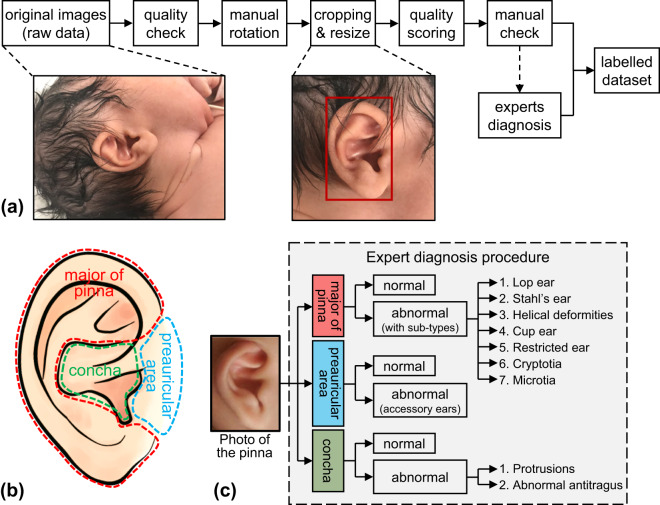
Procedure of image processing and labelling in BabyEar4k dataset. (**a**) gives a sketch of the image processing procedure. The raw ear photographs were rotated, cropped, and labelled to obtain the final dataset. (**b**) demonstrates the three regions of interest of the pinna, and (**c**) shows the diagnosis procedure according to the division of the three regions.

A general workflow to process the images in the ‘BabyEar4k’ dataset is included in the illustration of Fig. 2a. Firstly, a quality check was conducted for the raw image data exported from the smartphone. Photos that are missing, or of very poor quality (e.g. blurring, blockage or missing of the auricle etc.) were excluded from the dataset. Then, the images were rotated manually, roughly keeping the ears upwards. Afterwards, rectangular boxes indicating the boundaries of the ears were marked manually, then the ears and the neighboring areas were cropped with a ratio of 1:1, with the ear taken about 2/3. Then the cropped images were rescaled to a uniform size of 800 by 800 pixles. Afterwards, a further manual verification was made. The final auricle images were stored in.jpg format (24 bits, RBG channels). After data cleaning, 3,852 valid images from 1,926 newborns remained.

A subjective assessment of the image quality was conducted, through a collaboration and discussion involving two of the the authors. Four aspects were examined, each with three different scores: 1 for good-quality images, 2 for moderate-quality images, and 3 for poor-quality images. The four aspects assessed were 1) blur & noise, 2) cover-up, 3) brightness & contrast, and 4) shooting angle (see Table 1). These four aspects represented the different facets of image quality, which were influenced by multiple factors, such as the lighting conditions, the camera settings, etc. A total score, theoretically ranges from 4 to 12, was calculated by simply summing up the four scores. The images were then divided into two categories, one ‘good-quality’ category (2081 images) with total score = 4, and the other ‘not-good-quality’ category (1771 images) with total scores larger than 4. These two categories could be used in different scenarios. For example, the ‘good-quality’ images can be used for analyzing the infant ear anatomical shapes, or training an AI classifier for auricle deformities. The ‘not-good-quality’ images are often seen in clinic, thus these images can be included for practical usage.

**Table 1:** Scoring System for the Images Quality Assessment.

Aspects	Scoring scale
**Blur & Noise:**	1. Good image quality with clear demarcation of substructures.
Assessment of image blurring or noise, making auricle substructures hard to distinguish.	2. Slight or moderate blurring or noise, but the substructures can still be easily distinguished.
	3. Severe blurring, borders are not clear, or the substructures are hard to recognize.
**Cover-up:**	1. Little or no covering up, with minimal influence.
The covering up of dirt, hair, or other objects.	2. Moderate covering up (~5%).
	3. Severe covering up (>10%).
**Brightness & Contrast:**	1. Good brightness and contrast, with clear representation of the substructures.
subjective assessment of the brightness and contrast of the image.	2. Slight but acceptable problems exist, such as over- or under-exposed, lack of contrast, and color temperature deviation.
	3. Poor image qualities, with at least one severe aforementioned problem.
**Shooting Angle**	1. Good shooting angle, all the substructures were shown clearly.
The camera shooting angle should roughly perpendicular to the pinna, so that different substructures are shown intactly	2. The substructures are intact, but the shoot angle is a little bit skewed.
	3. The shooting angle is too skewed so that at least one substructure is missing (not due to deformity).

### Auricular deformity diagnosis

Two experienced experts (one ENT doctor (author Y-Y Fu) and one pediatrician (author F Luo)) participated in the diagnosis of the auricular shapes. A standardized diagnostic process was designed to facilitate the classification of complex auricular deformities (see Fig. 2b,c). The auricular area was artificially divided into three smaller regions, the ‘preauricular area’, the concha, and the ‘major part of pinna’. The experts conducted separate evaluations and judgements of the three regions. For the major part of pinna, they should firstly judge whether it was normal or abnormal; if abnormal, they should further select its sub-types. For the preauricular area, only a two-class classification (normal vs. abnormal) for each was needed. For concha, a three-class classification was conducted. The abnormality of the concha is called ‘abnormal protrusions’ or ‘abnormal antitragus’. The abnormality in the preauricular area is due to redundant tissues called accessory ears. The abnormalities in the three regions can co-exist in one ear, and the auricular deformity is defined as “at least one of the regions being abnormal”. The current diagnostic process is quite strict that the minimal deformities were identified.

The two experts blindly diagnosed the auricles following the above process, merely depending on the images. If the diagnostic results from the two experts for one ear are different, a further evaluation and discussion by these two experts were conducted to determine the final consensus.

### Collection of health data

The health data of the babies and their mothers were also collected, including the gender of the babies, their weights, the gestational ages, their mothers’ ages, the gravidity and parity, the amniotic fluid properties, the delivery mode, the mothers’ healthy data (high blood pressure, diabetes, etc). Details of the health data are described in Data Records.

## Data Records

### Data description

The BabyEar4k dataset is open for public use, which is available at figshare^19^.The final released dataset includes 3,852 ear images of 1,926 newborns from 1903 mothers (including 23 pairs of twins), together with the diagnostic results from two experts, and the healthy data. The images and diagnostic results facilitate the training of neural networks for diagnosis of newborn auricular deformities. The health data may be beneficial to study the non-hereditary risk factors of the deformities. This dataset does not distinguish between the train set and the test set.

### Data format

The BabyEar4k dataset contains the ear images along with the diagnostic results, and the health data. The directory structures and file types of the released dataset are described in Table 2.

**Table 2:** Directory structure and file types of the BabyEar4k dataset.

Directory and file names	File types	Descriptions
./images/[babyID]_[X].jpg	JPEG image file	Cropped images of the newborns’ ears. [babyID] is the id of the baby; and [X] is ‘L’ or ‘R’, indicating the side of the ears
./bounding.csv	comma-delimiter text file (.csv)	An annotation file that contains the ear boundaries in each image
./diagnosis_result.csv	comma-delimited text file (.csv)	The diagnosis results from two experts, as well as their consensus results.
./health_data.csv	comma-delimited text file (.csv)	Health information of the babies and their mothers
./image_quality_assessment.csv	comma-delimited text file (.csv)	Subjective assessment/scoring of the image qualities

### Image data, bounding annotations, and scoring

All images are stored in the ‘./image/’ directory, named as [babyID]_[X].jpg, where [babyID] is an integer indicating the unique id of the newborn in the dataset, and [X] can be either ‘L’ or ‘R’ indicating the left or right ear, respectively.

The images are cropped from the original photos for privacy protection. No further adjustments of the hues or lightness have been made. All images were resized into 800 by 800 pixles, containing the intact auricle. The annotations of the ear bounding box were stored in ./bounding.csv, with the following variables:

- **image_pathname:** the image path and filename,
- **left_bound:** the index of the left boundary,
- **top_bound:** index of the top boundary,
- **right_bound:** index of the right boundary,
- **bottom_bound:** index of the bottom boundary.
  The quality scores of the images (recall Table 1) are stored in the file ‘./image_quality_assessment.csv’. The variables include:
- **image_pathname:** the image path and filename,
- **blur_noise:** assessment of image bluring and noise, with levels 1, 2, or 3.
- **coverup:** assessment of the aspect ‘Cover up’, with levels 1, 2, or 3.
- **brightness_contrast:** assessment of image brightness and contrast, with levels 1, 2, or 3.
- **shooting_angle:** assessment of camera shooting angle, with levels 1, 2, or 3.
- **total_score:** The total score of the image quality.

### Diagnostic results

The diagnostic data of the two experts and their consensus results are stored in./diagnosis_resuls.csv, with the following variables:

- **baby_id:** the unique id for the newborn
- **directory_L:** the pathname of the image file of the left ear
- **directory_R:** the pathname of the image file of the right ear
- **L01:** the diagnosis result of the left ear from the 1^st^ expert
- **R01:** the diagnosis result of the right ear from the 1^st^ expert
- **L02:** the diagnosis result of the left ear from the 2^nd^ expert
- **R02:** the diagnosis result of the right ear from the 2^nd^ expert
- **L_merge:** the consensus diagnosis result of the left ear
- **R_merge:** the consensus diagnosis result of the right ear

The diagnostic results are presented in the format of ‘*a* + *b* + *c*’, where *a*, *b*, and *c* are integers representing the three auricle regions previously described in Section 2.3 of the paper, as well as in Fig. 2b. The integer ‘*a*’ represents the diagnostic result of the major part of the pinna, *a* = 0 if the region is normal, and ‘*a*’ is nonzero if it is abnormal, the number of ‘*a*’ indicate the sub-types. For example, if *a* = 1, this ear is diagnosed as a lop ear (see Fig. 2c). The second integer ‘*b*’ represents the preauricular area. ‘*b*’ has only two choices, 0 if this region is normal, and 1 if accessory ears exist. The last integer ‘*c*’ represents the concha region. ‘*c*’ equals to 0 if no abnormality is found, *c* = 1 if concha abnormal protrusions exist, and *c* = 2 if the antitragus is abnormal.

### Health data

The health data of the newborns and their mothers are stored in./health_data.csv, with the variables and value descriptions presented in Table 3.

**Table 3:** The keys and descriptions of health data in the BabyEar4k dataset.

Variable name	Value type	Descriptions
baby_id	integer, start from 1	unique id for newborns in this dataset
gender	categorical string, ‘female’ | ‘male’	the gender of the newborn
twins	integer	indication of whether the baby is one of the twins. If the value is 0, the baby has no twin. If the value is not 0, the other twin has the same value with him/her.
weight_grams	integer	the weight of the baby, unit: [grams]
gestational_age	integer	gestational age of the baby, unit: [weeks]
other_deformities	string, ‘no’ or short description of the deformities	short descriptive of the other diagnosed deformities of the newborns
mother_age	integer	The mothers’ ages
gravidity	integer	the gravidity
parity	integer	the parity
delivery	categorical strings, ‘natural’ | ‘forceps’ | ‘C-section’	The delivery method of the babies, ‘natural’ for natural childbirth (vaginal delivery), ‘C-section’ for Caesarean section, and ‘forceps’ for the usage of forceps.
amniotic_fluid_amount	categorical strings, ‘less’ | ‘normal’ | ‘more’	the amount of the amniotic fluid
amniotic_fluid_turbidity	categorical number, 0 | 1 | 2 | 3	the turbidity of the amniotic fluid, 0 for normal and clean amniotic fluid, and 1,2,3 for degrees of turbidity
amniotic_fluid_bloody	bool string, ‘yes’ | ‘no’	whether the amniotic fluid is bloody
high_blood_pressure	bool number, 0 | 1	Whether the mother has high blood pressure, 1 for yes and 0 for no
anaemia	bool number, 0 | 1	Whether the mother has anaemia
diabetes	bool number, 0 | 1	Whether the mother has diabetes
thyroid_disease	bool number, 0 | 1	Whether the mother has dyfunctions of the thyroid, e.g. hypothyroidism

## Technical Validation

### Natural distribution of the ear deformities

**Fig. 3:**
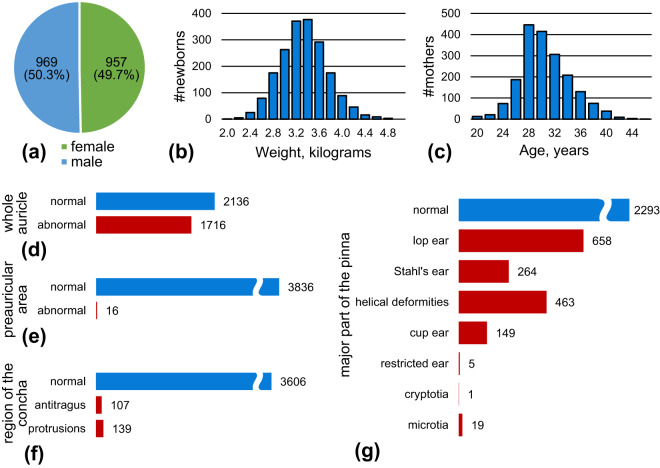
Data distribution properties of the dataset. (**a**) gives the gender distribution of the newborns. (**b,c**) plot the histograms of the newborns’ weight and the ages of their mothers. (**d-f**) gives the distribution of diagnosed auricular deformities, where (**d**) gives the evaluation of the whole auricle, and (**e**) to (**g**) draw the distributions of deformity sub-types of the three regions, i.e. the preauricular area, the concha, and the major part of the pinna.

To verify that the collected dataset has no gender, district or other types of bias (since all the data were collected in one center), statistical distributions of the data were calculated. The distributions of the babies’ genders, weights and the ages of the mothers are shown in Fig. 3a–c. The male-to-female ratio of newborns included in this study is close to 1. The average newborns weight is 3,337 ± 411 grams. The average age of the mothers is 31.0 ± 4.0 years. For 70.8% of the mothers, the newborns are their first babies.

Figure 3d–g and Table 4 gives the natural distribution of the auricular deformities. In total, 1,716 of the 3,852 ears (about 44.5%) have been evaluated as abnormal to some extent. The occurrence of abnormalities of the three auricle regions (major part of the pinna, preauricular area, and concha) are 40.5% (1,559 ears), 0.4% (16 ears), and 6.4% (246 ears) respectively. The most common sub-types of auricle deformations (major part of the pinna) are lop ears (17.1%, 658 ears), helical deformities (12.0%, 463 ears), and Stahl’s ears (6.9%, 264 ears). The occurrence of auricular deformities, as well as the distribution of their sub-types are consistent with previous researches^1,2^.

**Table 4:** Statistics of the auricular deformities in BabyEar4k dataset.

Regions	major of pinna		preauricular area		concha		the whole auricle	
Ears	normal	abnorm.	normal	abnorm.	normal	abnorm.	normal	abnorm.
Left ear	1126	800	1918	8	1809	117	1050	876
Right ear	1167	759	1918	8	1797	129	1086	1050
Both ears	2293	1559	3836	16	3606	246	2136	1926

Note: the whole auricle is evaluated as abnormal when any of the three regions is abnormal.

### The average ear

For a simple verification of meaningful classification of the ear deformity types used in this dataset, we calculated the “average ears” for the most commonly seen deformities, inspired by the well-known concept of “average face”^20^. The steps of calculating average ears are simple: (1) extract the ear from the images using the ear bounding information, (2) convert the ears into grayscale and resize it to 400 by 300 pixels; (3) flip the image if it is a right ear; (4) average all the images; (5) re-adjust the brightness and the contrast for better visualization. For a set of *N* images with the same size, denoted as $\{{G}^{k}\},k=\left[1,2,\ldots ,N\right]$, the average image *A* is defined as:

$${A}_{ij}={\left(\frac{{G}_{ij}-{G}_{min}}{{G}_{max}-{G}_{min}}\right)}^{\gamma },{G}_{ij}=\frac{1}{N}\mathop{\sum }\limits_{k=1}^{N}{G}_{ij}^{k}$$

where ${G}_{ij}^{k}\in \left[0,1\right]$ is the grayscale value of the *i*×*j* pixel of image *G*^*k*^, and *G*_*ij*_ gives the average of *N* images; *G*_*min*_ and *G*_*max*_ are the minimal and maximal pixel value in *G*_*ij*_; the parameter *γ* controls the contrast of the average image *A*.

**Fig. 4:**
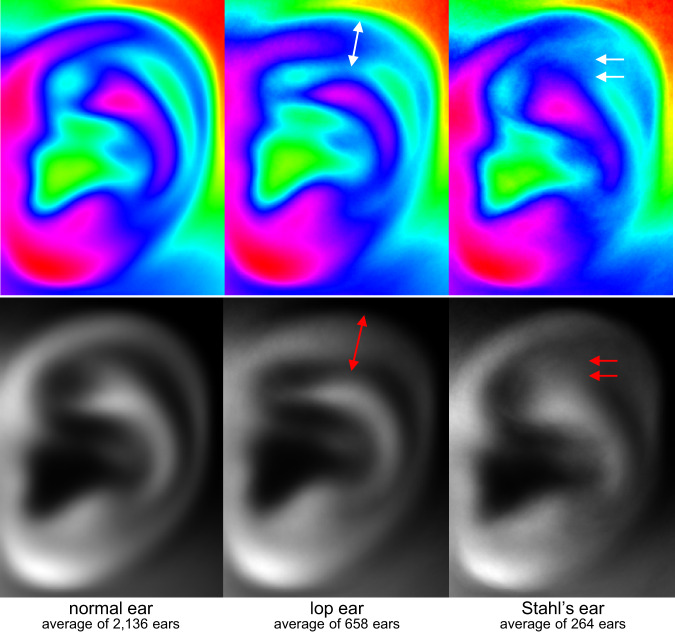
The average ears. From left to right are the normal ear, the lop ear, and the Stahl’s ear. The upper row gives pseudo-colored results (HSV colormap in Matlab), and the bottom row gives grayscale images.

Figure 4 gives the average ears of the normal ear, the lop ear, and the Stahl’s ear, respectively. Both grayscale (with γ=3) and pseudo-color (using HSV colormap in Matlab 2020a, with γ=1) images are shown. For the lop ears, the upper helix hangs down becomes thicker in the averaged image. For the Stahl’s ears, there exists a slight deformation at the upper helix, along with a new protruding crus. These changes are indeed the medically defined features of the deformative auricles^1,2^.

## Usage Notes

This dataset might be used for multiple purposes. Firstly, it serves as a medical database for analyzing the risk factors of auricle deformities. For example, male babies and early birth might be risk factors of ear deformities. Secondly, the images can serve as a dataset for anatomical analysis of ear shapes (especially deformities), whether using traditional image processing methods or neural networks.

Although the provided ear images are 3-channel RGB color images. We think that using a grayscale conversion might be a validated way to shrink the input data size, since the skin color is not so important in identification or classification of the auricular deformities. Besides, although we have rotated the ears manually, it is still recommended to rotate the image by randomly small angles during data augmentation.

While representing the natural occurrence and distribution of the auricular deformities, this dataset has some limitations mainly due to the significant differences in the incidence of each sub-types. Firstly, this dataset lacks enough data for some sub-types that rarely occurred, e.g. the congenial microtia (incidence of 1~4/10,000), cryptotia, and restricted ears (which can be regard as a severe type of cup ears). Secondly, the dataset ignores a typical sub-type called ‘protruding ear’. The sub-structures of the protruding ear could be well developed, with no deletion or deformation, except that the angle between the pinna and the head is abnormally larger. The protruding ear is not hard to distinguish if the 3D shape of the auricle is available. However, it is really hard, or impossible to identify this deformity merely relies on a 2D photograph. Therefore, in this dataset, we did not include this sub-type of deformities.

Nevertheless, this dataset is still useful for tasks such as distinguishing abnormal newborn ears from normal ones, or recognizing the commonly seen deformative sub-types. One of our future work is to enlarge the dataset to include more data of those rarely seen auricular deformities.

## Data Availability

The Matlab code for calculating the average ears are available at: https://www.github.com/willowfly/babyEar4k/.
